# Supplementary material for: Awareness, treatment, and control among adults living with arterial hypertension or diabetes mellitus in two rural districts in Lesotho
Source: PLOS Glob Public Health. 2024 Sep 30;4(9):e0003721. doi: 10.1371/journal.pgph.0003721 (PMC11441678; doi:10.1371/journal.pgph.0003721)
Supplement: S1 Checklist — (PDF) [file pgph.0003721.s001.pdf]

Inclusivity in global research

PLOS' policy on inclusivity in global research aims to improve transparency in the reporting of research performed outside of researchers' own country or community and ensures that PLOS publications reporting global research adhere to high standards for research ethics and authorship. Authors of relevant research articles may be asked to complete the questionnaire below, which outlines ethical, cultural, and scientific considerations specific to inclusivity in global research. This questionnaire may be requested when researchers have travelled to a different country to conduct research, if research uses samples collected in another country, research with Indigenous populations or their lands, or if research is on cultural artefacts. Researchers travelling to another country solely to use laboratory equipment will not normally be required to complete the questionnaire. However, the questionnaire can be requested at the journal's discretion for any submission – if you have been requested to complete this questionnaire by the PLOS journal you submitted to, please do so.

Please complete the questionnaire below and include this as a Supporting Information file with your manuscript. Note that if your paper is accepted for publication, this checklist will be published with your article in the supporting information files. Please ensure that you reference the checklist in the main body of your manuscript. We suggest adding a subsection 'Inclusivity in global research' to your Methods section and adding the following sentence: "Additional information regarding the ethical, cultural, and scientific considerations specific to inclusivity in global research is included in the Supporting Information (SX Checklist)"

The questions have been designed to be applicable to a wide range of study types, and there are subsections for both human subjects research and non-human subjects research. If any of the questions are not relevant to your research please mark them as "N/A" as appropriate.

Ethical considerations, permits and authorship

This section is applicable to all research types.

Provide details as to who granted permissions and/or consent for the study to take place in the Methods section of your manuscript. This should include the names of **all** ethics boards, governmental organizations, community leaders or other bodies that provided approval for the study. If individuals provided approval refer to these people by their role or title but do not list their name(s).

Reported on page number: Paragraph "Ethics statement" on page 7

If there were any deviations from the study protocol after approval was obtained please provide details of these changes in the Methods section of your manuscript.

Reported on page number: NA

Did this study involve local collaborators that are residents of the country where the research was conducted or members of the community studied? If you do not have any authors from said communities, please provide an explanation for this below.

Yes: TIL and RG co-conceptualized the survey, together with LGF/EF and AA/NDL. TIL is a MSc and soon PhD student from Lesotho, supported by this project. RG is project manager for SolidarMed, full-time based in Lesotho. LGF and EF are PhD students (from Spain & Nigeria), affiliated to the Division of Clinical Epidemiology and supported by this project. AA and NDL are co-PIs, who acquired the funding. MT and IA provided important technical input as part of their function (District Ministry of Health and SolidarMed Country Director). MPS, MK, MM, and MB are from Lesotho and collected the data in the field under the supervision of RG and with support from LGF and EF, who were part-time in Lesotho. The ComBaCaL survey team consisted of additional local part-time lay counsellors; they are acknowledged in the acknowledgments. Regular meetings between the team on the ground (MP, MK, MM, MB, RG, TIL, LGF, EF, IA) and the project management team members in Switzerland (AA, NDL) ensured that everyone was involved, and the survey could be adapted to the local needs and challenges.

Everyone listed as an author should meet PLOS' criteria for authorship and all individuals who meet these criteria should be included in the author byline, rather than the acknowledgements. For further information please see the journal's Authorship Policy.

Human subjects research (e.g. health research, medical research, cross-cultural psychology)

Did you obtain written informed consent from a representative of the local community or region before the research took place? How did you establish who speaks for the community? Details of written informed consent obtained from study participants should be reported separately in the Methods section of your manuscript.

The ComBaCaL project has official support from the local Ministry of Health and a community representative – both are represented in the Steering Committee and are official partners on the grant. Our local implementation partner (SolidarMed) has extensive experience in community engagement and is well connected to all community councils and the culturally needed processes involved for such a process. In short, all urban/rural community councils (council of village chiefs who represent further village chiefs) in both districts were consulted before the survey started and verbal proxy consent was obtained for all 120 village-clusters. On the day before the campaign the village chief to be visited the following day were contacted and, again, verbal proxy consent was obtained to be able to visit their villages. During the household visits, first verbal consent from the household head or representative was obtained, to visit their household and ask questions about/to their household members. Then, individual written informed consent was obtained from each participant.

How did members of the local community provide input on the aims of the research investigation, its methodology, and its anticipated outcome(s)?

This study is part of a 5-year large-scale project to evaluate and improve care for non-communicable diseases in Lesotho (ComBaCaL; www.combacal.org). The ComBaCaL steering committee includes a community representative, representatives from the Ministry of Health Lesotho, project leaders and researchers from the Lesotho-based non-governmental organisation and main implementer (SolidarMed) and researchers from the Division of Clinical Epidemiology University of Basel, Switzerland. The steering committee convenes regularly and defined the priorities for the ComBaCaL project with further input from the Ministry of Health. A key priority for the local partners of the Steering Committee was to assess the prevalence of arterial hypertension and diabetes mellitus in the project area and their care gaps, hence, we analysed the treatment care cascades presented in this manuscript.

During the piloting of the survey procedures we also held community gatherings to receive additional input from a wider community audience.

When engaging with the local community, how did you ensure that the informed consent documents and other materials could be understood by local stakeholders?

All survey material and especially the informed consent forms are reviewed by our local team members (TIL, MPS, MK, MM, and MB) and piloted.

Will the findings of the research be made available in an understandable format to stakeholders in the community where the study was conducted (e.g. via a presentation, summary report, copies of publications, etc.)? Please provide details of how this will be achieved.

Our local implementation partner (SolidarMed) regularly disseminates findings from all their projects such as ComBaCaL to local stakeholders at community gatherings, local health care and district health ministry team meetings, as well at national research fora at the Ministry of Health and its technical working groups.

In addition, we collaborate with another local partner (The HUB) to produce dissemination material such as: https://www.combacal.org/story
