# Supplementary material for: Awareness, treatment, and control among adults living with arterial hypertension or diabetes mellitus in two rural districts in Lesotho
Source: PLOS Glob Public Health. 2024 Sep 30;4(9):e0003721. doi: 10.1371/journal.pgph.0003721 (PMC11441678; doi:10.1371/journal.pgph.0003721)
Supplement: Attachment — Submitted filename: PGPH-D-24-00714 comments.docx [file pgph.0003721.s003.docx]

This population-based survey investigated the levels of awareness, treatment, and control regarding hypertension and diabetes in Lesotho. The study is particularly intriguing due to its identification and illustration of notable gaps within the hypertension and diabetes care cascade. However, I have some comments to add.

**Major comments**

1. Study setting: What factors influenced the choice of Butha-Buthe and Mokhotlong for the survey? Is there any other important characteristics beyond population size and hospital availability. Were considerations such as the prevalence of hypertension and diabetes, healthcare accessibility, demographic composition, existing healthcare infrastructure and resources, availability of trained professionals, and community participation, taken into account?
2. Study participants: What was the number of participants who had both hypertension and diabetes? Additionally, could you describe the characteristics of the cascade within this subgroup?
3. Discussion: What factors do the authors believe contribute to the disparity in awareness, treatment, and control between hypertension and diabetes?
4. Discussion: The findings indicated a lower number of individuals aware of their condition than those receiving treatment, with the authors attributing this difference to self-reporting. However, there may be additional factors influencing this, such as participants who are on medication but unaware that they have diabetes. Could this be linked to the health literacy or educational level of the population, or the patient education during treatment care?
5. Discussion: What factors could explain why individuals in urban or peri-urban areas had lower odds of awareness and control compared to those in rural areas? Typically, urban areas are expected to have better access to diagnosis and treatment, so why did this trend differ in the study results and contradict from previous articles?
6. Discussion: Are there existing gaps in Lesotho's NCD strategies, and how might this study influence policy changes? Please elaborate more on how the findings could be implemented in practice?

**Minor comments**

1. In page 4, line 113, please also cite the main survey article here.
2. The percentage for the age group 30-59 in the diabetes cascade within Table 1 should be corrected to 40.6%.
