# Supplementary material for: Awareness, treatment, and control among adults living with arterial hypertension or diabetes mellitus in two rural districts in Lesotho
Source: PLOS Glob Public Health. 2024 Sep 30;4(9):e0003721. doi: 10.1371/journal.pgph.0003721 (PMC11441678; doi:10.1371/journal.pgph.0003721)
Supplement: Attachment — Submitted filename: CBL_treatment_gap_PLOSGPH_20240723_pbp.docx [file pgph.0003721.s004.docx]

**Response to Reviewers**

Dear Editor and Reviewers,

Thank you for reviewing our manuscript titled **“Awareness, treatment, and control among adults living with arterial hypertension or diabetes mellitus in two rural districts in Lesotho”.** We appreciate your comments and efforts to improve the manuscript.

Find below our point-by-point responses to the comments raised. Our responses are in blue color.

**Reviewer #1:**

This population-based survey investigated the levels of awareness, treatment, and control regarding hypertension and diabetes in Lesotho. The study is particularly intriguing due to its identification and illustration of notable gaps within the hypertension and diabetes care cascade. However, I have some comments to add.

We would like to thank the reviewer for the thorough review.

**Major comments**

**1. Study setting: What factors influenced the choice of Butha-Buthe and Mokhotlong for the survey? Is there any other important characteristics beyond population size and hospital availability. Were considerations such as the prevalence of hypertension and diabetes, healthcare accessibility, demographic composition, existing healthcare infrastructure and resources, availability of trained professionals, and community participation taken into account?**

The study setting was chosen as these two districts fall under the implementation area of the ComBaCaL project (<https://www.combacal.org/>), where the main implementation partner (SolidarMed) is active and well connected to the community and local Ministry of Health. ComBaCaL is a five-year implementation research program developing innovative care models by training and equipping Community Health Workers to deliver screening, diagnosis and care for various chronic diseases. The first step of the programme was to conduct a baseline survey on the prevalence of various chronic diseases and their care cascades. The prevalence data has been published separately as part of this manuscript: <https://academic.oup.com/inthealth/article/16/3/313/7245757>

Our research consortium consisting of the Ministry of Health Lesotho, SolidarMed and the Division of Clinical Epidemiology Basel have successfully been collaborating in research for more than a decade and have a solid understanding of the local setting. Specifically on community participation, the ComBaCaL project was designed together with the ComBaCaL steering committee that includes a community representative, as well as representatives from the Ministry of Health of Lesotho. Furthermore, the study was discussed with local authorities such as village chiefs and community councils, who were engaged throughout.

We have added more context to section “Study setting”, page 4:

*This study is part of a population-based, cross-sectional survey conducted in the districts of Butha-Buthe and Mokhotlong, in Lesotho, from 1^st^ November 2021 to 31^st^ August 2022. The study was nested within the Community-based Chronic Care Lesotho Project (ComBaCaL; [www.combacal.org](http://www.combacal.org)). The first part of the project included a large population-based, cross-sectional survey assessing different chronic diseases and cardiovascular disease risk factors among individuals living in the project implementation districts in northern Lesotho. The prevalence data have been published elsewhere.^20^ In this study, we report the full care cascades to complement the published prevalence data.*

And we have added the prevalence findings from the separate publication in the introduction, page 3, to provide more context:

*Recent estimates from Lesotho suggest a 21.6% prevalence of hypertension and a 5.3% prevalence of diabetes in adults of 18 years and older.^19^ However, recent data about awareness, treatment and control of hypertension and diabetes are scarce^20^ and are not routinely used to guide programmatic interventions.*

**2. Study participants: What was the number of participants who had BOTH hypertension and diabetes? Additionally, could you describe the characteristics of the cascade within this subgroup?**

Overall, 149 participants had both hypertension and type 2 diabetes, i.e. 149/229 (65%) participants with diabetes also had hypertension and 149/1305 (11%) participants with hypertension also had diabetes. We added this information to baseline table 1 and added it to the results section. Of the 149 participants, 97 (65%) self-reported prior BP/diabetes diagnosis, 95 (64%) self-reported to be on treatment for both, and 45 (30%) had controlled BP and diabetes. However, we think, keeping the two cascades separate is easier to understand and more informative due to people e.g. being on treatment for one but not the other disease.

**3. Discussion: What factors do the authors believe contribute to the disparity in awareness, treatment, and control between hypertension and diabetes?**

In the discussion, we have expanded the comment on the possible factors for disparity related to the awareness, treatment, and control between hypertension and diabetes; first paragraph, page 14:

*We found disparities in treatment and control between hypertension and diabetes with worse outcomes across the diabetes cascade. This may be attributed to different factors. For example, clinics may lack the necessary equipment for regular glucose monitoring, whereas basic tools for BP measurement are more available, or healthcare professionals may be more aware and knowledgeable about hypertension and its complications than diabetes.*

**4. Discussion: The findings indicated a lower number of individuals aware of their condition than those receiving treatment, with the authors attributing this difference to self-reporting. However, there may be additional factors influencing this, such as participants who are on medication but unaware that they have diabetes. Could this be linked to the health literacy or educational level of the population, or the patient education during treatment care?**

Indeed, it is possible e.g. for people with diabetes who are on medication to be unaware of their diabetes diagnosis. We can only speculate on this aspect, for example that patients with low health literacy or multimorbidity might not be fully aware of their diagnosis, the nature of diabetes, or the importance of their medication and lifestyle changes. Or it may be that family members are managing medication for a patient, leading to delegated care and the patient not being fully aware of their own health condition. We have added these considerations in the discussion, last paragraph, page 15:

*This is the reason why in the diabetes care cascade (Figure 2B) the proportion of participants being aware or recalling a previous diagnosis is lower than those who reported being on diabetes treatment. Thus, this data needs to be interpreted with caution and we decided not to include this step in the multivariable analyses. It is indeed possible that people with diabetes and taking antidiabetic treatment are unaware of their diagnosis, for example due to delegated care or polypharmacy due to multimorbidity.*

**5. Discussion: What factors could explain why individuals in urban or peri-urban areas had lower odds of awareness and control compared to those in rural areas? Typically, urban areas are expected to have better access to diagnosis and treatment, so why did this trend differ in the study results and contradict from previous articles?**

We agree with the reviewer that this is a rather surprising finding. Importantly, these results were only found among people with hypertension but not diabetes. We have added an additional paragraph in the discussion to reflect on this (second paragraph, page 14):

*However, contrary to previous studies which have suggested that awareness, and control tend to be higher in urban areas^51,52^, our study found that adults living in urban areas had lower odds of being on hypertension treatment or achieving blood pressure control. This was not observed for diabetes. We hypothesize that this could be due to longer waiting times, rushed consultations, and inadequate follow-up at urban health facilities. Additionally, urban residents may be more mobile than their rural counterparts. However, these are speculations and need further research.*

**6. Discussion: Are there existing gaps in Lesotho's NCD strategies, and how might this study influence policy changes? Please elaborate more on how the findings could be implemented in practice?**

We think this study provides necessary data for the monitoring of the hypertension and diabetes treatment cascades for the Lesotho NCD programme. The Lesotho NCD programme manager is part of the ComBaCaL consortium. We not only disseminate these findings as a publication, but also at the Ministry of Health Lesotho and the NCD programme to ensure the findings are informing the local monitoring. We expanded our conclusion paragraph on page 15 further:

*The cascades of care for hypertension and diabetes in Lesotho have considerable gaps, indicating that access and quality of services remain insufficient to ensure adequate health outcomes. This study provides a baseline for future routine monitoring of these indicators across age and sex groups to support the progress to achieving programmatic targets in this setting.*

**Minor comments**

**1. In page 4, line 113, please also cite the main survey article here.**

We have added this reference as suggested.

**2. The percentage for the age group 30-59 in the diabetes cascade within Table 1 should be corrected to 40.6%.**

Thank you for this remark, this has been corrected.

----------------------------------------------------------------------------------------------------------------

**Reviewer #2:**

Thank you for the opportunity to review this paper

**General comments:**

**1. Will like to congratulate the authors for the work done and the willingness to get their study published. Well done!**

Thank you for the thorough review and positive remark.

**2. Was keen to know why authors are not interested in reporting prevalence based on their chosen methodology for which they could have increased the sample size especially for the participants with diabetes**

We agree with the reviewer that we did not state the aim of the study clearly enough. In fact, this study was part of a large population-based prevalence survey and these results were published separately (<https://doi.org/10.1093/inthealth/ihad058>). The aim of this study is to complement the prevalence findings with the care cascade. We have clarified the aim and design in the first paragraph of the Methods section, page 4:

*This study is part of a population-based, cross-sectional survey conducted in the districts of Butha-Buthe and Mokhotlong, in Lesotho, from 1^st^ November 2021 to 31^st^ August 2022. The study was nested within the Community-based Chronic Care Lesotho Project (ComBaCaL; [www.combacal.org](http://www.combacal.org)). The first part of the project included a large population-based, cross-sectional survey assessing different chronic diseases and cardiovascular disease risk factors among individuals living in the project implementation districts in northern Lesotho. The prevalence data have been published elsewhere.^20^ In this study, we report the full care cascades to complement the published prevalence data.*

And we have added the prevalence findings in the introduction, page 3:

*Recent estimates from Lesotho suggest a 21.6% prevalence of hypertension and a 5.3% prevalence of type 2 diabetes in adults of 18 years and older.^19^ However, recent data about awareness, treatment and control of hypertension and diabetes are scarce^20^ and are not routinely used to guide programmatic interventions.*

As such the sample size for this study was determined by the sample size of the main survey to obtain prevalence estimates.

**3. I have a fundamental challenge with the definition of diabetes and the definition of control of diabetes in the study**

Please find our response below, comment #10

**4. Generally, language and grammatical error should be checked.**

Thank you for your comment, we have revised the manuscript regarding language and grammar.

**5. Not clear on how sample sizes were arrived at from the methodology. It should be clearly stated**

As stated above, the sample size for this study was determined by the sample size of the main survey that aimed at obtaining prevalence estimates. No specific sample size was conducted for the estimates reported in this study. We have modified the paragraph “Study population”, page 6, as follows:

***Study population and Sample size***

*Following our survey protocol for the prevalence estimates^19^, we included participants who were found to have hypertension, defined as SBP ≥140 mmHg and/or DBP ≥90 mmHg and/or who reported to be taking antihypertensive medication (self-reported or written in their health booklet). Regarding diabetes, we included participants with confirmed diabetes, defined as RBG ≥5.6 mmol/l and HbA1c ≥6.5%, or being on antidiabetic treatment, or RBG ≥11.1 mmol/l in the absence of an HbA1c measurement. We used HbA1c in addition to establish the diagnosis, because the remoteness of the survey made it difficult to sample two blood sugar measurements on two different days and many participants encountered might not fulfil the criteria for fasting blood glucose. HbA1c has been recommended as alternative to fasting blood glucose for diagnosis of diabetes with high specificity in community settings.^28,29^The sample size for this study was determined by the sample size of the overarching survey that aimed at obtaining prevalence estimates and published elsewhere.^19^*

**6. Discussion needs more revision to make the study meaningful**

Thank you for your insightful suggestions, we have revised the discussion accordingly (see below).

Introduction
**7. “Lesotho has a population just over 2 million and is classified as a lower-middle-income country16.’ This statement should be part of the methods instead as readers get an idea of the study setting.**

We have transferred this statement to the methods section.

**8. If authors prefer to describe hypertension as ‘arterial hypertension” then its preferable to be more complete at systemic arterial hypertension or just keep as hypertension might even suffice.**

We have changed to “hypertension” throughout, as suggested.

**9. Might be helpful for readers to know the prevalence of hypertension and diabetes in Lesotho or as well as the burden of disease to make stronger case for the study.**

We have added the figures of prevalence in the Introduction, we refer to comment #2.

Methods
**10. The definition of diabetes with random blood glucose of >5.6 and Hba1c > 6.5% may be low and the definition of HbA1c of less than 8.0% for diabetes controlled is quite high. What is the source of this definition? It should be clearly stated and referenced. What about those with previous HbA1c of less than 8.0 already at diagnosis. Will they be considered good control if on medications?**

Participants on medication were considered controlled if HbA1c was below 8.0%. We acknowledge that there are different options to derive a confirmed diabetes diagnosis. We opted for HbA1c to confirm the diagnosis of diabetes because the remoteness of clusters made it difficult to sample two blood sugar measurements on two different days and many participants encountered during the survey might not fulfil the criteria for fasting blood glucose. HbA1c has been recommended as alternative to fasting blood glucose for diagnosis of diabetes with high specificity in community settings:

Kaur G, Lakshmi PVM, Rastogi A et al.  Diagnostic accuracy of tests for type 2 diabetes and prediabetes: a systematic review and meta-analysis. *PLoS One*. 2020; 15 (11): e0242415. <https://journals.plos.org/plosone/article?id=10.1371/journal.pone.0242415>

World Health Organization. *Use of glycated haemoglobin (HbA1c) in the diagnosis of diabetes mellitus. Abbreviated report of a WHO consultation*. Geneva: World Health Organization; 2011.

<https://iris.who.int/bitstream/handle/10665/70523/WHO_NMH_CHP_CPM_11.1_eng.pdf>

Both, the diagnostic criteria and the treatment control criteria are in line with similar surveys across 28 low-/middle-income countries:

Manne-Goehler, J. et al. Health system performance for people with diabetes in 28 low- and middle-income countries: A cross-sectional study of nationally representative surveys. PLoS Med 16, e1002751 (2019).

<https://journals.plos.org/plosmedicine/article?id=10.1371/journal.pmed.1002751>

We clarified the points above in the Methods section, included these references and adapted the second paragraph, page 6 as follows:

*Following our survey protocol for the prevalence estimates^19^, we included participants who were found to have hypertension, defined as SBP ≥140 mmHg and/or DBP ≥90 mmHg and/or who reported to be taking antihypertensive medication (self-reported or written in their health booklet). Regarding diabetes, we included participants with confirmed diabetes, defined as RBG ≥5.6 mmol/l and HbA1c ≥6.5%, or being on antidiabetic treatment, or RBG ≥11.1 mmol/l in the absence of an HbA1c measurement. We opted for HbA1c to confirm the diagnosis of diabetes because the remoteness of the survey made it difficult to sample two blood sugar measurements on two different days and many participants encountered might not fulfil the criteria for fasting blood glucose. HbA1c has been recommended as alternative to fasting blood glucose for diagnosis of diabetes with high specificity in community settings.^28,29^*

**11. Participants characteristics - Enrolment of participants is detailed in figure 1. “In the 120 clusters, the study teams visited 3498 households with 7412 eligible household members and identified 1308 participants with arterial hypertension and 291 participants with diabetes as per survey definitions.’ If sample size was well calculated enough prevalence of DM and HPT in Lesotho could be reported. Can authors work on this?**

As mentioned above, this study was nested in the main prevalence survey, published elsewhere (<https://doi.org/10.1093/inthealth/ihad058>). We are aware that there is some overlap (in the methods, not data), however, we think it is important to provide the information about the entire study sample selection again here.

**12. There is supposed to be a figure of study flow but can’t be seen on page 9 but its legend. Authors should please rectify.**

The figures have been uploaded separately.

**13. What informed the varying sample size, the authors should explain vividly in the methods**

We kindly refer to our answer regarding question #5.

Results
**14. Study is reporting very high awareness rate and high control rates for both DM and hypertension.**

We agree with this interpretation and have mentioned this in the discussion.

**15. Authors should have studied a few more variables like family history of the convictions. Risk factors such as smoking and alcohol intake, sedentary lifestyle etc. as well as complication of the diabetes and hypertension – this made the discussion quite hollow considering the good data collected.**

Thank you for this comment. The complication data have been published separately (<https://pubmed.ncbi.nlm.nih.gov/37883005/>). Similarly, we have published a manuscript with substance use and mental health data including information on smoking and alcohol (<https://link.springer.com/article/10.1007/s11469-024-01309-w>), and have explored traditional CVD risk factors with diabetes and hypertension prevalence in the main survey publication (<https://doi.org/10.1093/inthealth/ihad058>). We think that the data of traditional CVD risk factors are more relevant for the prevalence estimates than the care cascades.

Discussion
**The discussion needs a lot of tightening considering the study conducted. Major revision required with the discussion. Some few examples as a guide.**

We made several adaptations to the discussion and hope this meets the reviewers’ expectations.

**16. “Our results show insufficient rates of awareness, treatment, and control for both conditions. With regards to hypertension, one in three adults did not know they had hypertension, nor had initiated treatment, and only half of all the adults with hypertension had adequate BP control.”**

**Considering the relatively good awareness and control rate I think author should begin the discussion with that opening statement that compares to normal referenced estimates.**

We adapted the discussion by highlighting the rather high awareness and control rates, as well as adding the global 80-80-80 targets as a reference:

*Our findings indicate that the gaps along the hypertension and diabetes care cascades in Lesotho are lower than the regional average^28–30^. However, taking the 80-80-80 global targets for the diabetes and hypertension care cascades as a reference^31,32^, our results still show insufficient rates of awareness, treatment, and control for both conditions.*

**17. “Our study found no significant association between household wealth and awareness, treatment initiation, or control. How does this compare to other studies? What could be the possible reasons? That should be discussed thoroughly.**

We expanded on this result, added more references, and a hypothesis for our findings.

The lack of association between household wealth, treatment initiation and control for hypertension and diabetes may be explained by the fact that chronic care services are quite homogeneous in Lesotho with a minimal out of pocket fee, ensuring similar opportunities to initiate and maintain treatment, regardless of wealth. However, further research and analyses are needed to explore this finding. Last paragraph, page 13:

*The authors suggested that these gaps were heavily influenced by out-of-pocket expenses, i.e., patient fees for services, as indicated by studies from South Africa^41,42^ and multi-country analyses^30^. Our study found no significant association between household wealth and treatment initiation or control. In Lesotho, services for hypertension and diabetes are predominantly free in health centers, and patients are only required to pay a minimal fee when attending hospital services. Our results may be and indication of less inequality of chronic care services in Lesotho, providing similar opportunities to initiate and maintain treatment regardless of wealth. However, this study was not specifically designed to address this question and further research is needed to explore this association.*

**18.” In our study, we did not find a significant association between participants living with HIV and outcomes across the hypertension or diabetes care cascades.” Wondering why authors are rather interested in HIV and its association with DM and hypertension? Authors should focus more on the traditional risk factors or modifiable factors for diabetes and hypertension.**

As mentioned above, we have explored the traditional CVD risk factors with regards to the prevalence estimate in a previous publication (<https://doi.org/10.1093/inthealth/ihad058>) and think they are more relevant for the prevalence than the care cascade. However, an interesting finding from neighboring settings is that people living with HIV may have better outcomes along the hypertension/diabetes care cascades due to integrated service delivery. We wanted to explore such associations. We have clarified this point and added more details:

*A growing body of evidence fosters the idea that HIV treatment programs can successfully integrate, and thus strengthen, care for CVDs in settings with a HIV prevalence such as Lesotho^33,43–45^ For example, a recent study reviewing hypertension and diabetes outcomes along the HIV care cascade in rural South Africa found that participants living with HIV had lower systolic BP and blood glucose than participants not living with the virus^43^. We did not find a significant association between participants living with HIV and better outcomes across the hypertension and diabetes care cascades.*

Comment from the authors:

We merged the Patient and Public Involvement with the Inclusivity with Global Research statement in the Methods (page 8) and added the PLOS Checklist as a Supplement.
